# CRISPR/Cas9-Targeted Disruption of Two Highly Homologous *Arabidopsis thaliana DSS1* Genes with Roles in Development and the Oxidative Stress Response

**DOI:** 10.3390/ijms24032442

**Published:** 2023-01-26

**Authors:** Ivana Nikolić, Jelena Samardžić, Strahinja Stevanović, Jovanka Miljuš-Đukić, Mira Milisavljević, Gordana Timotijević

**Affiliations:** Laboratory for Plant Molecular Biology, Institute of Molecular Genetics and Genetic Engineering, University of Belgrade, Vojvode Stepe 444a, 11042 Belgrade, Serbia

**Keywords:** *AtDSS1*, oxidative stress, CRISPR/Cas9 technology, *Arabidopsis thaliana*, protein homeostasis

## Abstract

Global climate change has a detrimental effect on plant growth and health, causing serious losses in agriculture. Investigation of the molecular mechanisms of plant responses to various environmental pressures and the generation of plants tolerant to abiotic stress are imperative to modern plant science. In this paper, we focus on the application of the well-established technology CRISPR/Cas9 genome editing to better understand the functioning of the intrinsically disordered protein DSS1 in plant response to oxidative stress. The Arabidopsis genome contains two highly homologous DSS1 genes, *AtDSS1(I)* and *AtDSS1(V)*. This study was designed to identify the functional differences between *AtDSS1s*, focusing on their potential roles in oxidative stress. We generated single *dss1(I)* and *dss1(V)* mutant lines of both Arabidopsis *DSS1* genes using CRISPR/Cas9 technology. The homozygous mutant lines with large indels (*dss1(I)del25* and *dss1(V)ins18*) were phenotypically characterized during plant development and their sensitivity to oxidative stress was analyzed. The characterization of mutant lines revealed differences in root and stem lengths, and rosette area size. Plants with a disrupted *AtDSS1(V)* gene exhibited lower survival rates and increased levels of oxidized proteins in comparison to WT plants exposed to oxidative stress induced by hydrogen peroxide. In this work, the *dss1* double mutant was not obtained due to embryonic lethality. These results suggest that the DSS1(V) protein could be an important molecular component in plant abiotic stress response.

## 1. Introduction

DSS1 (deletion of split hand/split foot 1) is a highly conserved acidic eukaryotic protein consisting of a short chain ranging from 70–90 amino acids and a mass of 7–9 kDa, depending on the species. The DSS1 protein or SHFM1 (split hand/split foot malformation type 1) was primarily linked to the human developmental syndrome characterized by the absence and/or fusion of certain fingers [1]. As an intrinsically disordered protein, DSS1 interacts with components of multiple protein complexes and participates in diverse biological functions due to its flexible and non-defined three-dimensional structure [2,3,4,5,6]. DSS1 from different species has an alpha helix at the C-terminus end and two conserved acidic regions with a poorly conserved linker between them [3]. DSS1 protein without a helix complements the *Schizosaccharomyces pombe dss1* mutant phenotype; accordingly, most bindings to DSS1 occur in the disordered region [7]. Its wide range of functions is manifested in its participation in the ubiquitin-26S proteasome system [8,9,10], the breast cancer 2 (BRCA2)-DNA repair complex [11,12,13], the pre-mRNA splicing complex [14] and the transcription-export-2 (TREX-2) complex [15]. In budding yeast, the suppressor of exocyst mutations 1 (Sem1), the ortholog of human DSS1, establishes a greater number of interactions with proteins that contain the proteasome, COP9 signalosome, eukaryotic translation initiation factor 3 (PCI) domain, such as proteins of 26S proteasome, constitutive photomorphogenesis 9 (COP9) signalosome and the eukaryotic translation initiation factor 3 (eIF3) [14,15]. Importantly, PCI-domain-containing proteins are necessary for post-transcriptional gene and protein regulation [16].

In the light of proteostasis, Sem1 is the smallest regulatory and structural subunit of the 26S proteasome where it participates in the proteasome assembly. It contributes to the stability of the 19S proteasome lid by the successive recruitment of the regulatory particle non-ATPase 3 (Rpn3) and Rpn7 lid subunits [8,17,18]. While proteasome assembly is possible, it is not fully functional in the absence of Sem1 because Sem1 is necessary for Rpn7 modulation for efficient ATP-dependent substrate unfolding during proteolysis [19].

In the homologous recombination (HR) repair pathway, the most accurate mechanism in double-strand break repair, mammalian DSS1 interacts with the predominant mediator, the tumor suppressor protein BRCA2. DSS1 blocks BRCA2 multimerization and stabilizes the BRCA2 monomer through major structural rearrangements [13]. Overall, DSS1 is necessary for the proper activity of this recombination mediator protein [20,21,22]. In addition, the small acidic DSS1 protein has been shown to mimic DNA and to reduce the affinity of the replication protein A (RPA) for single-stranded DNA in exchange for RAD51 loading to single-strand DNA during repair [23]. In the HR machinery, DSS1 is likely to form protein complexes that do not contain PCI domains [7].

Sem1 is part of a large system that is important for transcription and pre-mRNA splicing during nuclear export. It was suggested that Sem1 as a subunit is involved in two structurally related complexes with a distinctive function in mRNA processing. The first complex is the ternary Thp3-Csn12-Sem1 complex, which mediates pre-mRNA splicing. Thp3 tethers to the Csn2-Sem1 binary complex where Sem1 strongly connects only with the Cop9 signalosome complex subunit 12 (Csn12) subunit [24,25]. The second complex is the nuclear mRNA-export complex that is built by Sac3-Thp1-Sem1. Although Sem1, as a single chain, binds primarily to Thp1, it establishes a weak contact with Sac3. Tethering between the human homologs Sem1 (DSS1) and Thp1 (PCID2) was also confirmed. It appears that Sem1 has a stabilizing role on Scn12 and Thp1 that facilitates complex assembly [25]. Once again, it is proved that Sem1 stabilizes the PCI domain-containing proteins and promotes complex assembly.

Interestingly, besides the well-known role of the DSS1 protein in 26S proteasome constitution, where it serves as a linkage between proteasome building blocks, it has been revealed that this molecule also interacts with the oxidized proteins [26]. Namely, in the context of a high reactive oxygen species (ROS) level and molecular damage, DSS1 plays a role in damaged protein labelling, thus leading to their degradation through the ubiquitin-proteasome system. This novel post-translational modification that leads to recognition and removal of oxidized protein was named DSSylation [26]. However, no data on the role of DSS1 and DSSylation in plant response to oxidative stress are currently available. Thus, our aim was to investigate the contribution of two Arabidopsis DSS1 proteins in maintaining protein homeostasis and to elucidate their potential role in prevention of the accumulation of toxic protein aggregates during oxidative stress.

Noteworthy, the Arabidopsis genome contains two highly homologous genes, *DSS1(I)* (At1g64750) and *DSS1(V)* (At5g45010), located in chromosomes I and V, respectively. The best-studied role of plant DSS1 proteins is their involvement in DNA repair mediated by BRCA2-driven HR [27]. There are also two isoforms of BRCA2 in Arabidopsis that can bind two DSS1 proteins, DSS1(V) interacts only with the AtBRCA2 (V) protein, whereas DSS1(I) interacts with both BRCA2(V) and BRCA2(IV) [27]. Studies have shown that Arabidopsis DSS1 proteins are independently associated with both the TREX-2 complex and the 26S proteasome. It is assumed that AtDSS1 proteins potentially link these two complexes when it is necessary to bring them into physical proximity [28]. As was shown in other species, one of the two Arabidopsis homologs, DSS1(V), has been identified as an mRNA nuclear export interaction partner and thus as a TREX-2 component [28].

In the functional analysis of *DSS1* genes, several *dss1* mutants from different species have been generated and described phenotypically. While *sem1* mutants of yeast [29], *dss1* mutants of *Ustilago maydis* [12] and *dss-1* mutants of *Caenorhabditis elegans* [30], have been characterized, information about plant *dss1* mutants is insubstantial. Only the T-DNA *Atdss1(V)* mutant with intron insertion has been characterized so far [31].

Understanding the possible functional dissimilarity of highly homologous genes in the genome of certain organisms is a challenge. A widely used efficient and precise gene editing strategy based on the clustered regularly interspaced short palindromic repeats-associated protein 9 system (CRISPR/Cas9) is routinely applied in experimental models from bacteria to mammalian cell lines and is also easily adapted for plant genome modification [32]. The CRISPR/Cas9 strategy has several advantages in plant technology, from the examination of gene functions to the improvement of crop varieties [33,34,35]. Carefully designed single guide RNA (sgRNA) ensures specific targeting, even when the genome contains closely related paralogous genes [36].

In this paper, we analyzed the structural differences between DSS1(I) and DSS1(V) as well as their interactions with putative protein partners in silico. The approach for the creation and selection of mutations in these two highly similar genes via the *Agrobacterium*-mediated CRISPR/Cas9 genome editing system is also reported. The generated mutant lines were phenotypically characterized and compared to wild-type (WT) plants during plant development. In addition, the present study shows the results of the examination of the susceptibility of both Arabidopsis *dss1* mutants to oxidative stress and of different levels of oxidized proteins between them.

## 2. Results

### 2.1. In Silico Comparative Analysis of DSS1(I) and DSS1(V) Proteins and Prediction of Their Specific Interactomes

**Figure 1:**
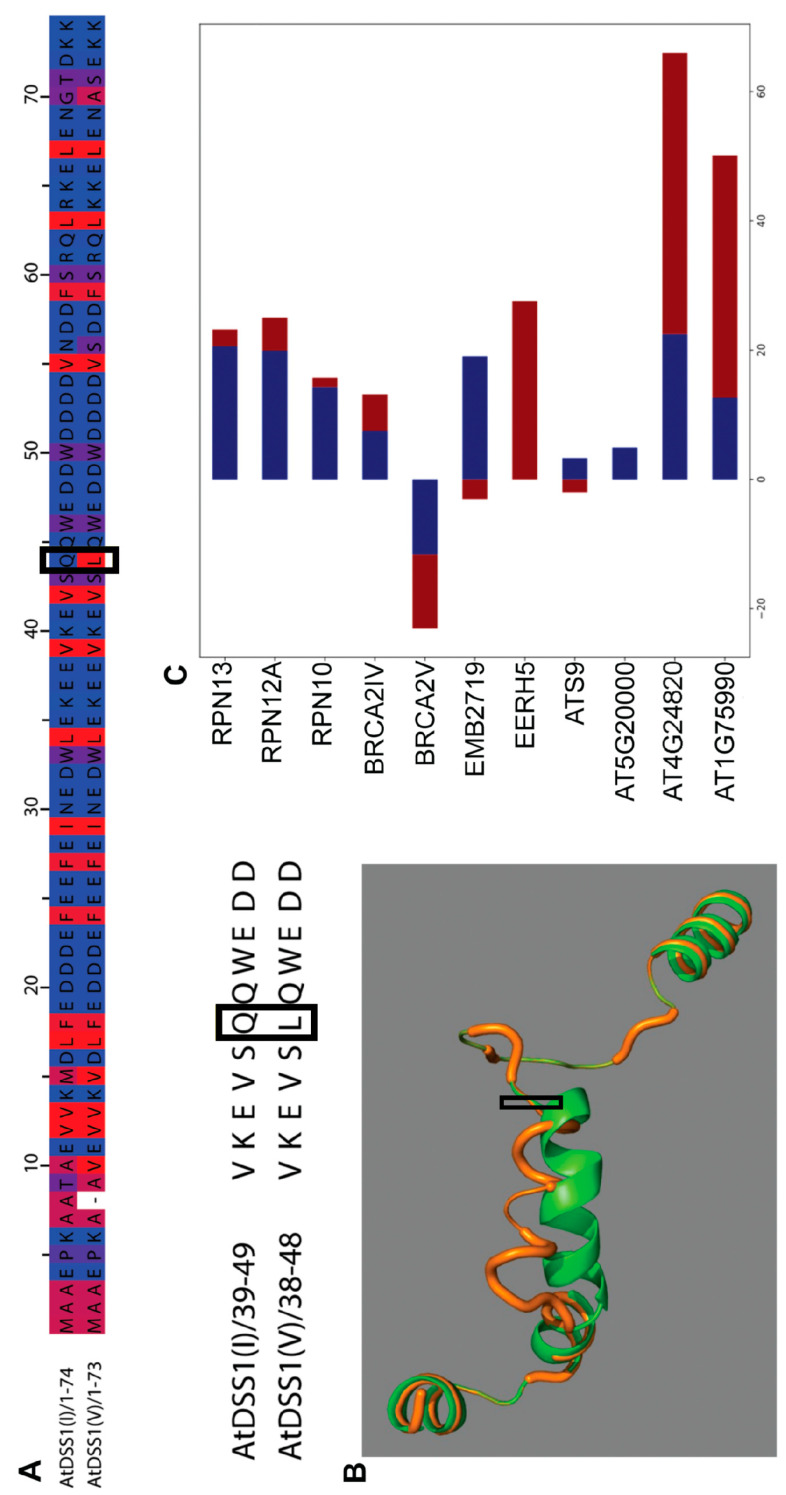
In silico DSS1 protein analysis. (**A**) DSS1(I) and DSS1(V) primary amino acid sequence alignment colored by hydrophobicity: hydrophobic—red, hydrophilic—blue; (**B**) visual comparison of DSS1(I) and DSS1(V) proteins using superimposed 3D protein models obtained using PHYRE V 2.0; black squares depict Q44L; (**C**) Binding energy of the solution in kcal/mol, given for specific partners. Blue and red represent the potential interaction of DSS1(I) and DSS1(V), respectively. The PPI (protein-protein interaction) properties were calculated using PatchDock and FireDock.

We analyzed and compared the primary amino acid sequences of DSS1(I) and DSS1(V) using the PSIPRED algorithm, and the alignment is shown in Figure 1A, where blue and red are the hydrophilic and hydrophobic parts, respectively. A remarkable similarity was noted between these two isoforms: DSS1(V) is only one amino acid shorter and contains seven different amino acids in comparison to DSS1(I). Out of these seven alterations, only one represents a significant change in amino acid polarity—from glutamine to leucine at position 44 (Q44L) (Figure 1A, yellow square). Potential differences in folding between DSS1(I) and DSS1(V) isoforms due to amino acid changes are shown in Figure 1B. The mutation annotated as Q44L could also distinguish the polarity properties between DSS1 types, which could contribute to different protein folding.

Due to the intrinsically disordered nature of DSS1 proteins, the docking results were insufficient to accurately explain the differences between DSS1(I) and DSS1(V) in interactions with their protein partners. However, a comparison of the protein–protein interaction between DSS1s and their potentially different partners may be useful in elucidating the evolutionary nature of the effect of a single amino acid change. Figure 1C shows the energy requirements of DSS1s to bind to respective potential partners. The length of the bar correlates with the energy needed for the interaction. Interactions of DSS1(I) are shown in blue and of DSS1(V) in red. According to the binding energies, DSS1(I) protein binds more easily to RPNs and EMBRYO DEFECTIVE 2719 (EMB2719, AT1G20200) but with a lower affinity to CHLOROPLAST HEAT SHOCK PROTEIN 70-1 (AT4G24280) and the PCI/PINT associated module (PAM) domain protein (AT1G75990). The binding of both DSS1 types to BRCA2B and non-ATPase subunit 9 (ATS9) appears to be energetically similar (Figure S1). In addition, in terms of binding energies, only enhanced ethylene response 5 protein (EERH5, AT2G19560) could bind DSS1(V), whereas it was predicted that the AAA-type ATPase family protein (AT5G2000) could be an exclusive DSS1(I) partner (Figure 1C).

### 2.2. CRISPR/Cas9 Based Mutagenesis of Two Highly Homologous DSS1 Genes

To date, only Arabidopsis *dss1(V)* was subjected to mutant analysis and partial characterization. Homozygous T-DNA insertional *dss1(V)* mutant plants were obtained and they showed a 75% reduction in the level of gene expression. Physiologically, the mutants displayed a significant reduction in rosette size and shoot length during growth, as well as a higher sensitivity to oxidative stress [31]. However, since two gene copies, *DSS1(I)* and *DSS1(V)*, are present in the Arabidopsis genome, single genetic mutants need to be generated for appropriate functional analyses.

**Figure 2:**
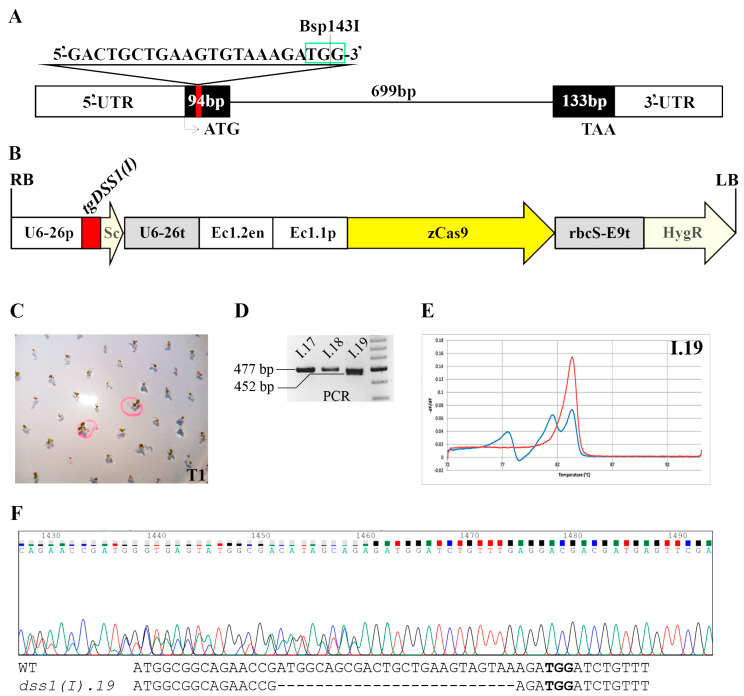
Construction strategy for *AtDSS1(I)* gene editing by CRISPR/Cas9 via *Agrobacterium*-mediated transformation of Arabidopsis and mutant screening approaches. (**A**) Schematic diagram of the *AtDSS1(I)* gene indicating the sgRNA target site (*tgDSS1(I)*) (red line), its sequence with PAM (green square), and Bsp143I restriction site; 5’ and 3’ UTRs are shown as white boxes; exons are shown as black boxes; the line indicates intron position; ATG is start codon and TAA is stop codon. (**B**) Structure of the CRISPR/Cas9 binary vectors pHEE401E for Arabidopsis transformation: *tgDSS1(I)* was controlled by the U6-26p–U6 promoter for PolIII; Sc-scaffold; the Cas9 cassette was driven by the EC1.2en and EC1.1p—egg-specific enhancer-promoter combination for zCas9 (*Zea mays* codon-optimized Cas9) expression in egg and zygote; rbcS-E9t—terminator of zCas9; Hyg—hygromycin-resistance gene; RB/LB are T-DNA right/left borders. (**C**) The photo of the selection of hygromycin-resistant Arabidopsis seedlings in the T1 generation. (**D**) The electropherogram of DNA fragments amplified by the primers designed to encompass the potential indel site (plant I.19 with large deletion (*del*); I.17, I.18—examples of plants with same PCR fragment length as WT plant). (**E**) HRM analysis of *dss1(I).19* genotype. (**F**) Sanger sequencing chromatogram of the generated heterozygous *Atdss1(I).19* plants in T2 generation, followed by the alignment of WT and mutant sequence. Bold letters represent PAM sequence.

**Figure 3:**
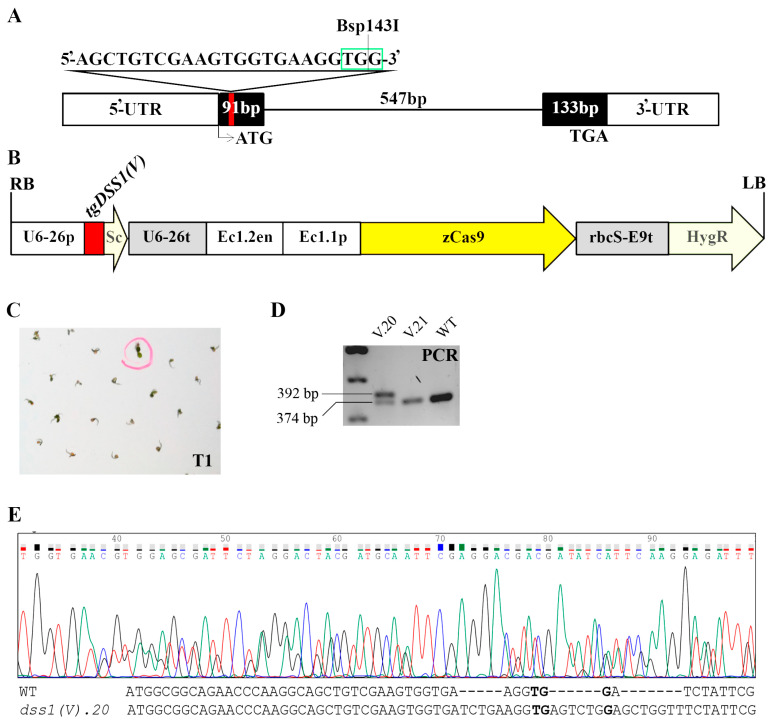
Construction strategy for *AtDSS1(V)* gene editing by CRISPR/Cas9 via *Agrobacterium*-mediated transformation of Arabidopsis and mutant screening approaches. (**A**) Schematic diagram of the *AtDSS1(V)* gene indicating the sgRNA target site (*tgDSS1(V)*) (red line), its sequence with PAM (green square), and Bsp143I restriction site; 5’ and 3’ UTRs are shown as white boxes; exons are shown as black boxes; the line indicates intron position; ATG is start codon and TGA is stop codon. (**B**) Structure of the CRISPR/Cas9 binary vectors pHEE401E for Arabidopsis transformation: *tgDSS1(V)* was controlled by the U6-26p–U6 promoter for PolIII; Sc-scaffold; the Cas9 cassette was driven by the EC1.2en and EC1.1p—egg-specific enhancer-promoter combination for zCas9 (*Zea mays* codon-optimized Cas9) expression in egg and zygote; rbcS-E9t—terminator of zCas9; Hyg—hygromycin-resistance gene; RB/LB are T-DNA right/left borders. (**C**) The photo of the selection of hygromycin-resistant Arabidopsis seedlings in the T1 generation. (**D**) The electropherogram of DNA fragments amplified by the primers designed to encompass the potential indel sites (plant V.20 with large insertion (*ins*); V.21—example of plant with same PCR fragment length as WT plant). (**E**) Sanger sequencing chromatogram of the generated heterozygous *Atdss1(V).20* plants in T2 generation, followed by the alignment of WT and mutant sequence. Bold letters represent PAM sequence.

We designed two separate sgRNA oligonucleotide inserts targeting the Arabidopsis *DSS1(I)* and *DSS1(V)* genes and subsequently constructed vectors for CRISPR/Cas9 gene-editing. Mutagenesis of duplicated genes with almost identical and extremely short sequences and functional redundancy was a challenge. To predict suitable sgRNA sequences, we had to meet important requirements to obtain high-quality target guide sequences. Namely, gDNA had to be located in the coding region near the 5’-end of the genes, to contain a suitable protospacer adjacent motif (PAM) sequence for Cas9 recognition, to be highly specific to avoid off-targeting, and to contain specific restriction sites at the position of the expected mutation for rapid screening by restriction analysis. Two separate sgRNAs (sgRNA-*DSS1(I)* and sgRNA-*DSS1(V)*) were created to target the first exon of both *DSS1(I)* and *DSS1(V)* genes that span the sequence (Figure 2A and Figure 3A). The sgRNAs were separately assembled with CRISPR/Cas9 constructs using the binary pHEE401E vectors containing a *Zea mays* codon-optimized *zCas9* gene under the control of an Arabidopsis egg-cell-specific promoter fused with an egg-cell-specific enhancer (Figure 2B and Figure 3B). Progeny seeds (T1) of transformed plants (T0) were collected and transferred to agar plates containing hygromycin as a selective marker. A transformation frequency of at least 0.4–0.8% was obtained with seeds collected from only two pots of the infiltrated plants. The first generation of transformed plants (Figure 2C and Figure 3C) was grown and individual plants were self-pollinated to produce the second generation. As we could not find a suitable restriction site that would overlap with the site of a potential indel, the search for potential mutant plants required the analysis of a larger number of potential mutants, as well as several screening approaches. Genotyping of T2 was conducted using three different strategies: PCR fragment length analysis, restriction analysis of PCR products using Bsp143I enzyme (data not shown) and high melting resolution (HRM) PCR. During the search for CRISPR/Cas9-*DSS1(I)* mutant plants, we conducted the genotyping of tens of young plants by PCR analysis. Amplicons encompassing the CRISPR/Cas9 target site were analyzed and only one PCR product deviating from the expected WT length was detected (Figure 2D). The plant labelled as *dss1(I).19* had a fragment that was about 20 nt shorter, which was selected for further sequencing. The heterozygosity of *dss1(I).19* was confirmed by HRM analysis (Figure 2E), while Sanger sequencing revealed that plant lost 25 nucleotides in mutated allele (Figure 2F). The gene editing event resulted in the generation of a mutant line with the introduction of a premature stop codon.

The progeny of the hygromycin-resistant CRISPR/Cas9-*DSS1(V)* plant was subjected to PCR fragment length analysis (Figure 3D). PCR products encompassing the potential target site of mutagenesis in plant *dss1(V).20* contained an additional band, as a result of mutagenesis. Sequence analysis confirmed that this plant is a heterozygous mutant line containing an 18 nt insertion in the *DSS1(V)* gene (Figure 3E). Although the 18 nt long insertion represents an in-frame mutation, a premature stop codon was introduced within the mutated transcript. 

**Figure 4:**
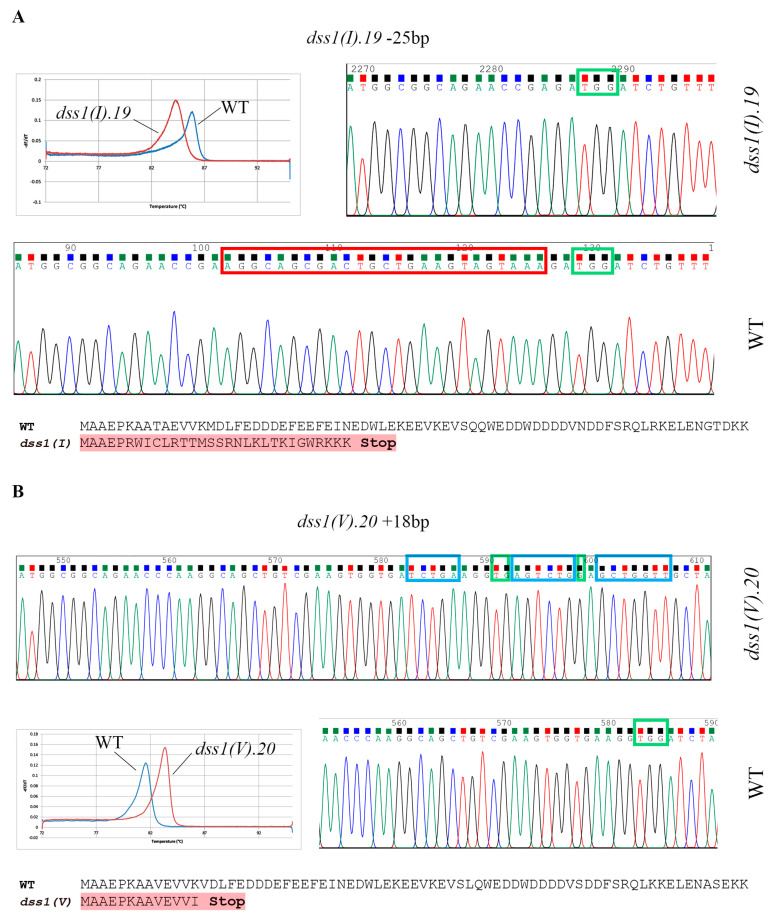
Mutation analysis of T3 generation. (**A**) HRM profiles, DNA-sequence chromatograms and amino acid sequences of the homozygous *dss1(I)* mutant and WT plant. (**B**) DNA-sequence chromatograms, HRM profiles, and amino acid sequences of the homozygous *dss1(V)* mutant and WT plant. Green squares represent PAM sequence, red square marks 25 nt of WT sequence that are deleted in mutant line named *dss1(I).19*; blue boxes mark 18 nt of insertion in *dss1(V).20* mutant line. The derived protein sequences were obtained by using ExPASy Translate tool (http://web.expasy.org/translate/; accessed on 28 July 2022)).

Mutant lines with larger indels (*del*25 nt and *ins*18 nt) were selected for further characterization because these mutations were easy to trace in further generations. Plants homozygous for *dss1(I)* and *dss1(V)* were selected in the T3 generation by HRM, and their homozygosity was confirmed by sequencing (Figure 4). To confirm disruption of the *DSS1* genes, i.e., the absence of WT transcripts, forward primers were designed to overlap the site of the putative indels. Using conventional PCR, we confirmed the complete absence of specific *DSS1* transcripts in the obtained mutant lines (Figure S2). In order to show that mutant lines are T-DNA free, i.e., that they do not contain CRISPR/Cas9 cassette between T-DNA right/left border sequence, we performed PCR on gDNA isolated from *dss1(I).19* and *dss1(V).20* and primers that amplify 423 bp of U6-26p promoter sequence. The corresponding gel electrophoresis is presented as Figure S3 in the Supplementary material.

After backcrossing pure homozygous single mutants with WT plants and recovering the desired mutations to the homozygous stage, we proceeded to traditional crossing between *dss1(I)*^−/−^ and *dss1(V)*^−/−^ to generate double *dss1(I)*^−*/*−^*dss1(V)*^−*/*−^ mutants. Seeds obtained from pollination of *dss1(V)*^−*/*−^ pistils by *dss1(I)*^−*/*−^ pollen and vice versa were subjected to genotyping. Plants that were *dss1(I)*^−*/*−^*dss1(V)*^−*/+*^ and *dss1(I)*^−*/+*^*dss1(V)*^−*/*−^ were selected by PCR and this genotype was allowed to self-pollinate. More than hundreds of the produced seedlings were analyzed by HRM and none of the *dss1(I)*^−*/*−^*dss1(V)*^−*/*−^ genotypes were detected (data not shown). The inability to generate double mutant strains suggests that the functions of these highly homologous genes may be essential to the plant cell.

To confirm that certain sgRNAs do not introduce off-target mutations, HRM analysis of potential off-target sites was carried out using gDNA from WT and mutant plants as templates. The best ranked potential off-target sites that contained 3 bp or more mismatches were selected for validation (Table S1). The genomic DNA sequences surrounding the potential off-target sites were amplified by PCR using gene-specific primers (Table S2). PCR products were analyzed by Sanger sequencing, and no changes in potential off-targets were detected.

### 2.3. Phenotypic Characterization of Arabidopsis dss1(I) and dss1(V) Mutant Lines

**Figure 5:**
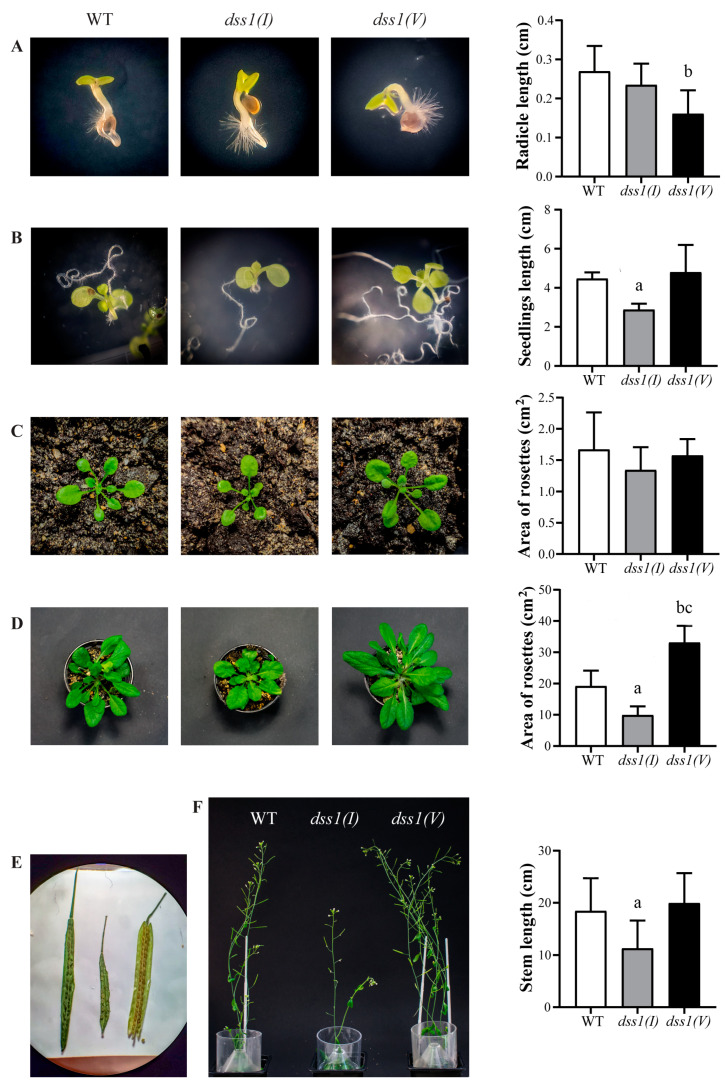
Phenotypic differences between Arabidopsis WT, *dss1(I)* and *dss1(V)* mutant lines during development. Seeds were cultured on the Murashige and Skoog (MS) solid medium under long-day conditions. After 2 weeks of growth in MS, all plants were transplanted into soil. (**A**) Images of 2-day-old WT, *dss1(I)*, and *dss1(V)* mutant plants grown on MS and histogram of radicle length in cm. (**B**) Images of 12-day-old plants grown in MS and histogram of seedling length in cm. (**C**) Images of 25-day-old plants grown in soil and histogram of rosette area in cm^2^. (**D**) Images of 7-week-old plants in soil and histogram of the rosette area in cm^2^. (**E**) Representative image of WT (left), *dss1(I)* (center) and *dss1(V)* (right) siliques. (**F**) Stems of 7-weeks-old WT, *dss1(I)*, and *dss1(V)* mutant plants in soil and histogram of stem length in cm. White bars correspond to quantifications of various parameters for WT, grey bars correspond to *dss1(I)* mutants, black bars correspond to *dss1(V)* mutants. Photographs show representative seedlings and plants. Data are presented as the mean ± SD of values obtained from three experiments (*n* = 30 per experiment). Significance of differences in histograms is labeled with lowercase letters; a, WT vs. *dss1(I)* (*p* < 0.05); b, WT vs. *dss1(V)* (*p* < 0.05); c, *dss1(I)* vs. *dss1(V)* (*p* < 0.05).

Plant phenotyping experiments aimed to reveal the correlation between the plant’s genome and its morphological traits. Monitoring was carried out throughout the different developmental stages (Figure 5). Plants were initially grown in MS medium for 2 weeks and then in soil until the end of the life cycle. In the early stage, slower germination of the mutant seedlings was observed compared to the rapid germination of WT controls (Figure 5A). In addition, WT seedlings had fully opened cotyledons, while mutant cotyledons were still closed at this developmental stage. Analysis of 12-day-old seedlings showed that the length of *dss1(V)* mutants was almost identical to that of the WT, but a significant difference in root length was observed between *dss1(I)* mutants and WT plants; the *dss1(I)* plants were 1.6-fold shorter (Figure 5B and Figure S4). At the post-sowing stage, a similar, but statistically non-significant trend continued among the three seedling genotypes on day 25 (Figure 5C). Subsequently, 7-week-old *dss1(V)* exhibited accelerated development and demonstrated a significant 1.7-fold increase when compared with WT rosettes. In contrast, *dss1(I)* plants showed a significant 1.9-fold reduction in growth compared with the WT (Figure 5D). The rapid growth of *dss1(V)* led to faster ripening and splitting open of most siliques and earlier seed release in comparison to WT plants. Compared to the WT, *dss1(I)* siliques were shorter and produced fewer seeds per silique (Figure 5E). Further measurements showed that *dss1(I)* stems were on average shorter than *dss1(V)* and WT (Figure 5F). Overall, the growth of *dss1(I)* was delayed compared to the WT, whereas *dss1(V)* developed faster than the WT.

### 2.4. Sensitivity of Atdss1(I) and Atdss1(V) Lines to Oxidative Stress

**Figure 6:**
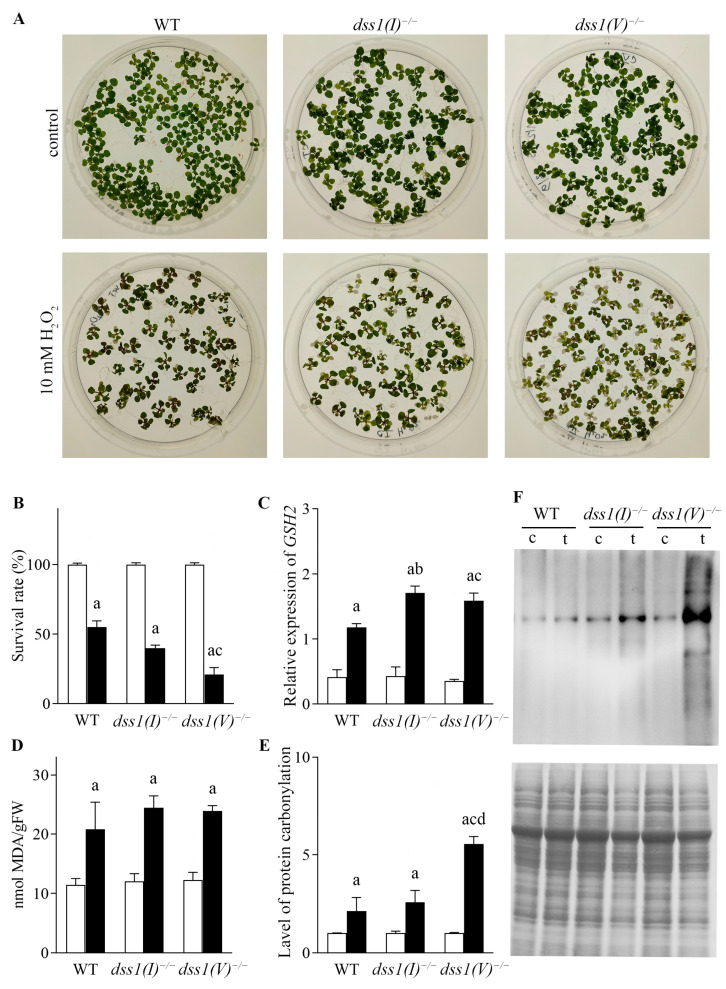
Effects of H_2_O_2_ stress on WT and CRISPR/Cas9 *dss1(I)^−/−^* and *dss1(V)^−/−^* mutant seedlings. 14-day-old MS-grown WT and mutant seedlings were transferred to control MS medium containing 10 mM H_2_O_2_. (**A**) Seedlings were grown for three days under stress condition before the images were taken. (**B**) Survival rates of Arabidopsis WT and *dss1(I)* and *dss1(V)* mutant seedlings grown under 10 mM H_2_O_2_. (**C**) Relative expression of *GSH2*. (**D**) Level of peroxidation (nmol/g FW) in WT and *dss1(I)* and *dss1(V)* mutant seedlings exposed to 10 mM H_2_O_2_ for one day. (**E**) Level of protein carbotilation (total band densities were determined by ImageJ software v1.53n for densitometry). (**F**) Determination of protein carbonylation: OxyBlot- (top) and Coomassie-Brilliant Blue (CBB) stained SDS-PAGE (bottom) of control (c) or 10 mM H_2_O_2_-treated (t) Arabidopsis WT or *dss1(I)* and *dss1(V)* mutant seedlings. Data shown are representative of three experiments; relative band density is presented as the total band density of carbonylated proteins detected by anti-DNP antibodies/band density of total proteins stained with CBB. In histograms white bars show control (c) and black bars show 10 mM H_2_O_2_-treated (t) Arabidopsis WT or *dss1(I)* and *dss1(V)* mutant seedlings. Experiments were performed in biological triplicates and data correspond to the mean ± SD. Significance of differences in histograms is labeled with lowercase letters; a, c vs. t (*p* < 0.05); b, WT vs. *dss1(I)* (*p* < 0.05); c, *WT* vs. *dss1(V)* (*p* < 0.05) and d, *dss1(I)* vs. *dss1(V)*.

Seeds of WT and mutated *dss1(I)* and *dss1(V)* lines were germinated in culture media containing 10 mM hydrogen peroxide (H_2_O_2_) (Figure 6). Chlorosis of young leaves in mutant lines treated with the stressors at the early stage of stress was more conspicuous than in WT plants (Figure 6A). To quantify the effects of the applied stressor, we determined the survival rate of the seedlings as the percentage of seedlings that germinated after the stress treatment compared to the control seedlings (Figure 6B). In the presence of H_2_O_2_, all three lines showed a significantly lower survival rate compared to the control seedlings. Interestingly, under H_2_O_2_ stress, *dss1(V)* plants had a 61% lower survival rate in comparison to WT plants under the same treatment, while the survival rate of *dss1(I)* seedlings was only 27% lower than the WT. In contrast to *dss1(I)* mutants, *dss1(V)* were clearly more sensitive to oxidative stress (Figure 6B).

In addition, the expression profiles of some stress responsive genes were analyzed, such as genes encoding the antioxidant enzymes glutathione synthetase (*GSH*) and catalase 1 (*CAT1*). The most significant differences were observed in the expression of the *GSH* gene (Figure 6C). According to our results, oxidative stress upregulated the expression of *GSH* in both *dss1(I)* and *dss1(V)* plants, as they had significantly elevated levels of *GSH* transcripts after H_2_O_2_ treatment in comparison to WT plants. Specifically, the level of the *GSH* transcript in the *dss1(I)* and *dss1(V)* mutant lines was 31% and 26% higher in comparison to the WT level of expression, respectively (Figure 6C). We found no statistically significant differences in the expression levels of the CAT1 gene among *dss1(I)*, *dss1(V)* and WT plants exposed to oxidative stress (data not shown).

We also quantified the malondialdehyde (MDA) content as a measure of cellular damage caused by oxidative stress. All three lines exhibited a 20–24% increase in MDA content under oxidative stress when compared with the WT, while under control conditions they showed no significant difference; however, no significant elevation of lipid peroxidation was detected in the mutant lines (Figure 6D). Finally, protein carbonylation, an irreversible oxidative protein modification, was measured by immunoblotting to determine the effects of oxidative stress. The amount of carbonylated products that accumulated in the mutant lines was greater in *dss1(I)* and *dss1(V)* plants than in WT plants. The most intensive oxidation was detected in the *dss1(V)* mutant line, where the amount of carbonylated proteins was 60% higher than in WT plants (Figure 6E,F). In the *dss1(I)* mutant line, only 18% more oxidatively damaged proteins were recorded than in WT plants. Our results suggest that *dss1(V)* mutants were more sensitive to oxidative stress than *dss1(I)* mutants as significantly more carbonylated products were detected in *dss1(V)* plants.

## 3. Discussion

It is well known that plants as sessile organisms are constantly exposed to different stimuli from the natural environment. Even small variations in environmental conditions, such as temperature fluctuations or lack of water, high salinity, exposure to heavy metals or radiation, produce an abiotic stress response in plants [37]. Environmental stressors trigger an exaggerated production and accumulation of ROS, which induce comprehensive plant stress response [38]. Molecules containing activated oxygen cause major cellular damage and dysfunctionality of all cellular compartments. Under conditions of oxidative stress, nucleic acids, lipids and proteins are oxidized and must be neutralized immediately to prevent their deleterious effects on cells. Diverse cellular antioxidant defense mechanisms have evolved to reduce oxidative damage in plant cells [39]. One of the most important goals of modern plant science is to identify new factors involved in the efforts of plants to maintain cellular homeostasis and overcome the effects of stress [40]. The results of intensive research on this topic can be used to improve agricultural productivity by sophisticated genetic engineering [41,42].

Our previous study on the role of plant DSS1 proteins indicated that protein and gene expression of plant DSS1 has an altered profile under the influence of different oxidative stressors [31]. Analysis of the Arabidopsis *dss1(V)^−/−^* T-DNA insertion mutant line showed increased sensitivity to oxidative stress, suggesting that it may be an important player in the molecular mechanisms of a plant’s abiotic stress defense [31]. The Arabidopsis genome contains two copies of the *DSS1* gene that differ slightly in length. *DSS1(I)* contains 222 bp whereas *DSS1(V)* contains 219 bp, encoding 74 and 73 amino acid-long protein isoforms, respectively [27]. Herein, we analyzed the primary amino acid sequences of DSS1(I) and DSS1(V) in silico and noted a remarkable similarity. DSS1(V) is shorter than DSS1(I) and differs in seven amino acids but with only one fundamentally significant change, from glutamine to leucine at position 44, which can affect protein structure and function. Considering that the primary amino-acid sequences of DSS1 orthologs in *S. pombe*, *S. cerevisiae* [8] and *C. elegans* [30] contain the polar amino acids threonine or asparagine at the same position in AtDSS1(I), it can be assumed that *AtDSS1(V)* is the result of gene duplication. About 65% of annotated genes in plant genomes are duplicated, and these genes are often resistant to the selective pressure to which they are normally exposed [43]. This can lead to an accumulation of multiple mutations in the sequences of the duplicated gene, potentially resulting in new functions [44]. The high percentage of homology between two Arabidopsis DSS1 proteins indicates that they may overlap in their functions and partner interactions. Since DSS1s are multifunctional proteins involved in diverse essential biological functions, plant DSS1s are likely to be functionally complemental in most of these functions. However, we hypothesized that minor, but significant changes in the primary structure of these extremely disordered proteins underlie the emergence of new functions.

Additionally, the comparison of protein–protein interactions between AtDSS1 proteins may also be useful in explaining the nature of the interactions and functional differences. According to our prediction, both isoforms generally have the potential to bind the same protein partners. However, we predicted that the distinctive predicted partner of DSS1(I) could be a protein encoded by locus AT5G2000, the 26S proteasome regulatory subunit 8 homologue B. The exclusive partner of DSS1(V) is the EER5 protein, which is involved in promoting a reduced ethylene response upstream or within the ethylene-activated signaling pathway. Accordingly, promoter analyses revealed the presence of ethylene-responsive elements in the *DSS1(V)* promoter sequence but not in the *DSS1(I)* promoter [31].

To ascertain other functional relationships between *DSS1* genes in cellular protein homeostasis during oxidative stress, we used the CRISPR/Cas9 approach for precise gene mutagenesis. The advantage of the Cas9 system is its capacity to simultaneously edit multiple loci in the same plant, enabling synchronized mutagenesis of multiple functionally related genes that belong to the same gene family [45]. We used CRISPR targeted mutagenesis for separate editing of two highly homologous *DSS1* genes. Although multiplexing the CRISPR/Cas9 gene editing approach is a strategy of choice for simultaneous and rapid mutagenesis, we used the traditional crossing method to generate double mutants. Our aim was to ensure the segregation of unwanted mutated loci by dissolving potential off-target mutations through traditional backcrossing with WT plants over several generations and restoration of the desired mutation to a homozygous stage.

Besides several mutants with small indels in each of the *DSS1* genes, we managed to select two lines with larger mutations that could be easily confirmed by genotyping. Two CRISPR constructs targeting different *DSS1* gene copies were generated and two homozygous mutant lines were selected for further characterization—a mutant line with a deletion of 25 nt in *DSS1(I)* and a line with an insertion of 18 nt in the *DSS1(V)* gene. CRISPR/Cas9-induced mutations occurred in both mutant lines and resulted in the generation of premature stop codons and protein truncation.

Several online prediction tools found some off-target loci in addition to the beneficial mutation(s) in *DSS1(I)* and *DSS1(V)* genes. However, CRISPR/Cas9 precision and off-target issues are not as problematic in plants as in animals. This is mainly because off-target mutations in plants can be successfully segregated and removed by backcrossing [46]. To prevent the undesired influence of random mutations on *dss1* mutant line phenotypes, backcrossing and further selection were conducted. Therefore, potential unwanted mutations were diluted and segregated over several generations. In addition, potential off-targets were screened by HRM PCR and Sanger sequencing.

Further characterization of Arabidopsis mutant lines with disruptions in each of the *DSS1* genes (*dss1(I)*^−*/*−^ and *dss1(V)*^−*/*−^) revealed visible phenotype dissimilarities in comparison to WT plants. Based on the phenotypic description, the differences between *dss1* CRISPR/Cas9 mutants suggest that they most likely have different cellular roles in addition to their overlapping functions, which we have already mentioned. The mature *dss1(I)* plants produced abnormal siliques and a very tiny habitus with only a few shoots at the fruit-ripening stage. The appearance of abortive seeds indicates a possible disruption in the homologous recombination that takes place in meiosis during sexual reproduction in Arabidopsis. For instance, the absence of AtBRCA2 is known to impair female gametophyte development [47]. Moreover, development of the *C. elegans dss-1* mutant germ line was slightly delayed and numerous oocytes were not produced [30]. Owing to the multifunctionality of *Atdss1(I)*, this aberrant regulation may not be exactly in meiosis, but in another DSS1 dependent mechanism required for oogenesis, combined with errors in HR. This suggests that the *AtDSS1(I)* transcript could be involved in the process of embryogenesis.

It is well known that the ethylene signaling pathway leads to cotyledon expansion when the seedling reaches the soil surface, as well as to silique maturation in the ripening developmental stage [48,49,50]. The presence of ethylene *cis*-responsive elements in the promotor region of the *DSS1(V)* gene indicates that this gene is likely an important player in the process of maturation. We assume that plants with a disrupted *DSS1(V)* gene have closed cotyledons, shorter primary roots in the early stage of development and faster maturing of siliques in comparison to WT plants, possibly because of the defective response of *dss1(V)* to ethylene.

Interestingly, while the *dss1(V)* mutant lines generated using CRISPR/Cas9 technology showed similar sensitivity to oxidative stress as the T-DNA insertional *dss1(V)* mutant plants from our previous work, significant and unforeseen phenotypical differences were noted. For example, mature plants with a T-DNA disruption of the *DSS1(V)* gene showed stunted growth, while CRISPR/Cas9 *dss1(V)* mutant plants on the contrary had a very bushy habitus with many shoots. This highly branched phenotype is consistent with the phenotype of the *brca2* mutant of Arabidopsis [51] and is not surprising, considering that both DSS1 and BRCA2 are from a complex that plays a key role in initiating this process [27,52,53]. On the other hand, the reason for the diversity of mutants in relation to different mutagenesis approaches could be explained by the partial cleavage of T-DNA together with the intron during the splicing process. Some expression regulators could be embedded within the introns; furthermore, some introns also contain promoter sequences for alternative transcripts [54,55,56]. Recent studies have also revealed that a large number of microRNAs are located within the introns of protein-coding genes [57,58,59,60]. Intron-derived microRNA mirtrons are alternative precursors for microRNA biogenesis and arise from the processing of gene introns [58]. Thus, the altered phenotype of the intron insertion mutant *dss1(V)* can be attributed to the disruption of both the *DSS1* gene and a potential regulatory molecule within the intron. This explains the importance and predictability of precise gene editing, which is made possible thanks to CRISPR/Cas9 technology.

We found that both single *dss1(I)* and *dss1(V)* mutants are viable and fertile, although the *dss1(I)* mutant line had reduced fertility. Our experiments showed that two gene mutations in *DSS1* genes are likely to be lethal when combined in a double mutant. When the pistils of *dss1(I)* mutant lines were hand-crossed with pollen donor mutant lines of *dss1(V)* and vice versa, analysis of the next generations revealed no double mutant genotypes among hundreds of analyzed young plants. This finding points to the importance of *DSS1* gene function as disruption of both gene copies clearly does not compromise viability. In addition, our results indicate that these two genes complement each other in essential biological functions.

Our findings indicated that *dss1(V)* mutant seedlings were more sensitive to oxidative stress induced by H_2_O_2_ in comparison to *dss1(I)* mutant seedlings. Although an increase in lipid peroxidation was not significantly different among analyzed genotypes, the survival rate of the *dss1(V)* line, but not the *dss1(I)* line, was markedly lower in comparison to WT counterparts under the stress condition. This implies that *dss1(V)* has an important role in plant stress defence. In addition, we noticed significantly increased *GSH2* gene expression in both the *dss1(I)* and *dss1(V)* lines in comparison to the WT line. Glutathione synthase 2 could be considered as one of the oxidative stress markers [61]. It regulates glutathione biosynthesis, especially in the catabolism pathways induced by endoplasmic reticulum stress [62]. We assume that DSS1 proteins could be involved in the organization of gene-regulatory machinery and, thus, in regulation of *GSH2* gene expression. Our assumption is supported by the fact that BRCA2-RAD51 complex, known for its function in HR, also plays a direct role in the regulation of defense gene transcription during plant immune responses [47]. On the other hand, DSS1-BRCA2 complex promotes RAD51 interaction with single stranded DNA, and therefore initiation of DNA-repair by homologous recombination [63]. Therefore, we assume DSS1 could be molecular glue that contributes to the functional integrity of multiple protein complexes involved in gene transcription. In addition, the most interesting finding was a dramatic increase in the level of oxidized proteins in *dss1(V)* mutant seedlings exposed to H_2_O_2_ in comparison to WT, but also to *dss1(I)* counterparts. This again implies that DSS1(V), in distinction to DSS1(I), has a role in oxidative stress response in Arabidopsis. Excessively accumulated oxidized proteins in *dss1(V)* mutant plants exposed to H_2_O_2_ stress indicate that *DSS1(V)* gene is one of the vital players in cellular detoxification from oxidatively damaged proteins and maintenance of protein homeostasis. Our results are in line with a novel DSS1-dependent post-translational protein modification reported in human cells, i.e., DSSylation [26].

In addition, we assume that Q44L substitution could contribute to the observed phenotypic differences between two DSS1 isoforms. One of the possibilities is that these two proteins differently contribute to the constitution of 26S proteasome and, consequently, to elimination of damaged proteins. The other is that these two proteins might have different affinity to bind oxidatively damaged proteins in the process of DSSylation. Indeed, our results corroborate such assumption, as exclusion of DSS1(V) led to inefficient elimination of oxidized proteins, unlike the disruption of DSS1(I) protein that had a limited effect on the process.

Our study was the first to demonstrate functional differences between *AtDSS1* genes and their influence on the plant phenotype and developmental dynamics. Two DSS1 protein isoforms have different effects on the maintenance of protein homeostasis, which could be attributed to the different sensitivity of mutant plants to oxidative stress in comparison to WT plants. These findings also emphasize the importance of developing efficient strategies for mutagenesis and are highly relevant for understanding the molecular mechanism of the plant response to oxidative stress.

## 4. Materials and Methods

### 4.1. Plant Material and Treatment

Arabidopsis plants were cultured in sterile Murashige and Skoog (MS) agar medium containing 1% sucrose (Suc) [64]. For sterile cultivation in MS + Suc plates adjusted to pH 5.7, seeds were surface-sterilized with 70% (*v*/*v*) ethanol for 5 min, followed by 20 min incubation in a sterilizing solution (10% commercial bleach, 0.05% (*v*/*v*) Tween 20 (Sigma-Aldrich, St.Loius, MO, USA). The seeds were washed 3 times with sterile distilled water (dH_2_O), resuspended in 0.1% (*w*/*v*) agarose and seeded on MS + Suc plates. After 5 days of dormancy at +4 °C, seedlings were grown on the plates for 7 days. For genotyping and phenotyping, Arabidopsis plants were grown in soil (Flora substrate mixed with sand at a 4:1 ratio, fertilized by watering with liquid fertilizer substrate 0.5% (*v*/*v*)) using the Arasystem (Ghent, Belgium). Plants were grown in a growth chamber under standard conditions as follows: a long-day regime (16 h day), light intensity of 150 μmol m^−2^s^−1^, relative humidity of 70%, day/night temperatures of 21 °C/19 °C. ImageJ open-source software was used to measure the root length, total leaf area and stem length [65]. Root lengths were determined both in vertical and horizontal plates. Measuring of the root lengths was performed by tracing the main root using “freehand lines” tool in the ImageJ software. To measure rosette area, a white square was added to the images for scaling. To analyze the stress response, Arabidopsis was grown on agar plates containing 10 mM H_2_O_2_. Seedlings were collected and immediately frozen in liquid nitrogen 3 days after the start of treatment. All samples were stored at −80 °C prior to use.

### 4.2. In Silico Analysis of Protein Structure

Protein–protein interactions were predicted using the STRING functional protein association network [66]. Coordinate files for homology models were determined using sequence identity criteria (average identity 37%). The DSS1 model structures were created using the Phyre2 protein folding recognition server by uploading the following sequences: AtDSS1(I) (Uniprot:AT1G64750) and AtDSS1(V) (Uniprot:AT5G45010) [67]. The best template structure, human 26S proteasome bound to ubiquitin carboxyl-terminal hydrolase 14 (USP14)-ubiquitin-like modifier-activating enzyme 1 (UbAl) (pdb:5gjg) has 66% identity with a query sequence [68]. After preparing the protein data bank (PDB) model, the docking protocol was applied using the PatchDock algorithm with a cluster root mean square deviation (RMSD) of 4 Å [69]. The top 20 cluster structures were subjected to FireDock refinement based on transformations [70]. The scoring function is based on geometric fitting and atomic desolvation energy [71]. The parameters used to calculate the solution global binding energy (GBE) are the atomic contact energy or desolvation energy for the two proteins at transit from the unbound state to the complex (ACE), hydrogen and disulfide bonds (HB) and aliphatic interactions (ALIPH), attractive and repulsive van der Walls forces, short- and long-range Coulomb forces, cation-π and π-π stacking interactions.

### 4.3. sgRNA Design

Appropriate sgRNA sequences were selected using two in silico tools: CHOPCHOP v2 [72] and CRISPR-P v2.0 [34] to target the *DSS1(I)* (acc. no. AT1G64750) and *DSS1(V)* (acc. no. AT5G45010) genes of *Arabidopsis thaliana.*

### 4.4. Construction of the CRISPR/Cas9 Binary Vector and Plant Transformation

According to the selected sgRNA for site specific editing of *AtDSS1(I)* and *AtDSS1(V)* genes, two pairs of oligonucleotides complementary to each other and containing tails composed of 4 nt from the BsaI restriction recognition site were synthetized by Metabion (Planegg, Germany): sgDSS1(I)f: 5’-ATTGGACTGCTGAAGTAGTAAAGA-3’; sgDSS1(I)r: 3’-CTGACGACTTCATCATTTCTCAAA-5’ and sgDSS1(V)f: 5’-ATTGAGCTGTCGAAGTGGTGAAGG-3’; sgDSS1(V)r: 3’-TCGACAGCTTCACCACTTCCCAAA-5’. For CRISPR/Cas9 editing we used pHEE401E binary vector. Plasmid pHEE401E (No. 71287) enable egg cell-specific promoter-controlled expression of 3 × FLAG-NLS-zCas9-NLS and it contains gRNA scaffold for insertion of target sequence controlled by U6-26 promoter, as well as hygromycin gene resistance. To clone guide oligonucleotides into the binary vector pHEE401E, the Golden Gate reactions containing 20 µM oligonucleotides (sgDSS1(I)f and sgDSS1(I)r or sgDSS1(V)f and sgDSS1(V)r, 200 ng of pHEE401E vector, 1 × bovine serum albumin and 5 U of BsaI (Restriction Endonuclease Products, New England Biolabs Inc. (NEB), (Ipswich, MA, USA) in 1 × T4 ligase buffer (NEB) were carried out. The reactions were incubated at 37 °C for 5 h, at 50 °C for 5 min and 80 °C for 10 min on a Biometra T1 thermocycler (Göttingen, Germany). The obtained vector was used for transformation of *E. coli* by heat-shock transformation [73]. The isolated plasmids (kit obtained from Qiagen, Hilden, Germany) were sequenced with the following primers: U6-26p-F 5’-TGTCCCAGGATTAGAATGATTAGGC-3’ and U6-26t-R: 5’-CCCCAGAAATTGAACGCCGAAGAAC-3’ by Macrogen Europe BV (Amsterdam, The Netherlands). The obtained pHEEK401E-*DSS1(I)* and pHEEK401E-*DSS1(V)* vectors were introduced into the C58C1 strain of Agrobacterium by electroporation. After electroporation, the Agrobacterium suspension was transferred to agar plates containing 50 μg·mL^−1^ kanamycin and incubated overnight at 28 °C. Arabidopsis plants were transformed by the floral dip infiltration method [74] using Agrobacterium C58C1 carrying the vectors pHEEK401E-*DSS1(I)* or pHEEK401E-*DSS1(V)*. Seedlings of *Arabidopsis thaliana* Col-0 seeds obtained from plants subjected to floral dip transformation were plated on 1% agar containing MS medium and hygromycin at a concentration of 50 μg·mL^−1^.

### 4.5. Genomic DNA Isolation

For genotyping, individual young leaves of Arabidopsis seedlings were placed in plastic tubes containing 400 µL of Extraction buffer (200 mM Tris-pH 7.5–8, 250 mM NaCl, 25 mM ethylenediaminetetraacetic acid (EDTA), 0.5% sodium dodecyl sulfate (SDS)). Leaf tissue disruption was carried out using stainless steel beads in the Tissue Lyser II (Qiagen, Hilden, Germany) 2× for 45 s at 30 Hz. After pelleting, the cellular debris by centrifugation at >10,000× *g* for 5 min, 300 µL of the supernatant was transferred to tubes containing 300 µL of isopropanol. The solution was mixed by inverting the tube several times and left at room temperature for 5 min. Samples were then centrifuged at full speed for 10 min and dried pellets were dissolved in 100 µL of sterile 10 mM Tris-HCl, pH 8, 1mM EDTA (TE buffer). Genomic DNA for analysis by sequencing was isolated using the DNeasy^®^ Plant Kit (Qiagen, Hilden, Germany).

### 4.6. Workflow for Screening Gene-CRISPR/Cas9-Modified Plants

To evaluate the mutations introduced into transgenic plants, the flanking sequence around the CRISPR/Cas9 target sites was amplified by PCR with gene-specific primers: ScsI f: 5’-TCTCGATCTGGTTGGTTCCT-3’/ScsIr: 5’-CCTATCTCACACCTGAAATTGACA-3’ and ScsVf: 5’-TCTCGATCTGGTTGATTTGCT-3’/ScsVr: 5’-ATCCGTTTTCGCATCAGAAC-3’, which were designed to amplify 477 bp and 374 bp of *DSS1(I)* and *DSS1(V)*, respectively. PCR was performed under the following cycling program: 5 min at 95 °C, 30 cycles at 95 °C for 45 s, at 61 °C for 30 s, 72 °C for 1 min and for 5 min at 72 °C. For PCR-restriction enzyme analysis, PCR-amplicons were used directly for digestion with the restriction enzyme Bsp143I (Sau3AI) (Thermo Fisher Scientific™, Waltham, MA, USA).

Prior to sequencing, HRM analysis was performed on diluted (2000×) PCR products of undigested Bsp143I DNA. Melting curves were analyzed with the MicPCR software Version 2.12.2 (Bio Molecular Systems, Brisbane, QLD, Australia), using a 5 × HOT FIREPol^®^ EvaGreen^®^ HRM Mix (Solis BioDyne, Tartu, Estonia). HRM was performed under the following cycling program: 15 min at 95 °C, 40 cycles at 95 °C for 15 s, at 60 °C for 20 s, at 72 °C for 20 s; a standard melting curve was generated with small temperature increments (0.1 °C/s) using a Mic Real Time PCR Cycler (Bio Molecular Systems, Brisbane, QLD, Australia). For fine HRM determination, insert-specific nested primers were designed to amplify smaller fragments: 105 bp of *DSS1(I)* and 103 bp of *DSS1(V)*, (ScaIf: 5’-TCTCGATCTGGTTGGTTCCT-3’/ScaIr: 5’-CTCATCGTCGTCCTCAAACA-3’ and ScaVf: 5’-TCTCGATCTGGTTGATTTGCT-3’/ScaVr: 5’-ACTCATCGTCGTCCTCGAAT-3’. PCR amplicons with melting curves different from WT melting curves were selected and sequenced by Macrogen Europe BV (Amsterdam, The Netherlands). The sequencing chromatograms were compared using Tide software (accessed on 13 Jun 2021) [75].

To show that mutant lines are T-DNA-free, the following primers were used to amplify U6-26p promoter: U6-26p-F: 5’-TGT CCC AGG ATT AGA ATG ATT AGG C-3’, and U6-26p-R: 5’-CCC CAG AAA TTG AAC GCC GAA GAA C-3’.

### 4.7. Off-Target Site Analysis

Potential off-target sites were searched using CHOPCHOP v2 and CRISPR-P v2.0 software, and low potential off-targets were detected with up to 2 mismatches. Primers designed to overlap the nominated potential off-target sites are listed in Table S2. Further, amplicon sequencing was performed to identify off-target cleavage events in selected regions.

### 4.8. RNA Isolation and cDNA Synthesis

Plant tissue was frozen in liquid nitrogen and ground using a mortar and pestle. RNA was isolated from 30 mg of powdered sample by the GeneJET RNA Purification kit (Thermo Fisher Scientific, Waltham, MA, USA) according to the manufacturer’s protocol. To remove any DNA remains prior to cDNA synthesis, total RNA samples were treated with the DNA-free™ DNase Treatment and Removal DNA Kit (Ambion^®^, Austin, TX, USA). cDNA synthesis was performed according to the Thermo Fisher Scientific protocol using a random hexamer primer and the RevertAid™ (Thermo Fisher Scientific, Waltham, MA, USA) reverse transcriptase.

### 4.9. PCR and Gene Expression

The following primer pairs were designed using Primer Express software (accessed on 14 December 2020) for the detection of *DSS1* transcripts: tdIf: 5’-CTGAAGTAGTAAAGATGGATCTGTTT-3’/tdIr: 5’-TGGCTAACTTCCTTCACTTCT-3’, and tdVf: 5’-AAGTGGTGAAGGTGGATCTATTC-3’/tdVr: 5’-CATTTCTTCTCACTAGCATTCTCAAG-3’. The primers are complementary to the first exon of the WT sequences of *DSS1* genes. PCR was performed under the following cycling program: 5 min at 95 °C, 35 cycles at 95 °C for 30 s, at 62 °C for 30 s, at 72 °C for 30 s and for 5 min at 72 °C.

For gene expression analysis, prior to the SYBR Green assay, total cDNAs were diluted 1:5 with nuclease-free water. Reactions were performed in 25 µL containing 300 nM of each primer and 2 × SYBR Green PCR Master Mix (Thermo Fisher Scientific, Waltham, MA, USA). Real-time PCR was conducted on the ABI Prism 7500 Sequence Detection System (Applied Biosystem, Waltham, MA, USA) under the following cycles: for 2 min at 50 °C, 10 min at 95 °C and 40 cycles at 95 °C for 15 s and 60 °C for 1 min. Each PCR reaction was performed in triplicate and no-template controls were included. Amplification of PCR products was detected in real time and the results were analyzed with 7500 System Software (Applied Biosystem) and presented as 2^−ΔCt^. For gene expression analysis of selected genes related to the oxidative stress response (AT5G27380 glutathione synthase 2 and AT1G20630—catalase 1), the following primers were used: GSH2F: 5’-ATTGGCTAAAGCTTGGTTGGAGTA-3’/GSH2R: 5’-CGTTCTTCTGGCTGTACAATTACCA-3’ and CAT1F: 5’ -AGGAGCCAATCACAGCC-3’/CAT1R 5’-TCAAGACCAAGCGACCA-3’. Actin was used as endogenous control and amplified by ActF: 5’-CTTGCACCAAGCAGCATGAA-3’ and ActR: 5’-CCGATCCAGACACTGTACTTCCTT-3’.

### 4.10. Lipid Peroxidation Measurement

The lipid peroxidation level in the samples was measured by determining the peroxidation product MDA [76]. One hundred mg of powdered plant tissue was homogenized in 800 µL of 20% trichloroacetic acid (TCA). After centrifugation at 14,000× *g* for 20 min, 600 µL of the supernatant was added to 600 µL of 20% TCA containing 0.5% thiobarbituric acid (TBA). After heating at 95 °C for 30 min, the mixture was cooled on ice for 5 min and centrifuged at 14,000× *g* for 10 min. Specific absorbance was measured at 532 nm and non-specific absorbance at 600 nm. MDA concentration was calculated using the extinction coefficient of 155 mM^−1^·cm^−1^ and expressed as MDA nmol per 1 g of fresh weight (FW).

### 4.11. Protein Isolation and Immunoblot Analysis

Pulverized samples (100 mg) were resuspended in 0.2 mL of buffer containing 25 mM Tris-HCl, 2 mM EDTA and 0.1 mM phenylmethylsulfonyl fluoride (PMSF). Samples were stored on ice for 60 min with periodical vigorous vortexing. Samples were centrifuged at 10,000× *g* for 15 min at 4 °C and the supernatants were transferred to new tubes. To verify the quality of the protein extracts, samples were analyzed by SDS-polyacrylamide-gel electrophoresis (PAGE) in 12% acrylamide. The gels were stained with 0.2% Coomassie Brilliant Blue (CBB).

### 4.12. OxyBlot Analysis

For protein gel blot analysis, equal amounts of protein (25 μg) were separated from total protein extracts isolated from Arabidopsis seedlings by 12% SDS-PAGE and transferred to a polyvinylidene difluoride membrane (PVDF) membrane (Millipore, Burlington, MA, USA) using the Fastblot B43 transfer system (Biometra, Analytik Jena, Germany) according to the manufacturer’s instructions. For the detection of carbonyl groups introduced into the protein by oxidation, the OxyBlot^™^ Protein Oxidation Detection Kit (Millipore, Burlington, MA, USA) was used. To inhibit protein oxidation during the protein extraction (as described above), 50 mM dithiothreitol (DTT) was added to the samples. About 25 µg of protein in 5 µL of each sample was denatured with an equal volume of 12% SDS. The carbonyl groups in the protein side chains were derivatized to 2,4-dinitrophenilhydrazone (DNP hydrazone) by 2,4-dinitrophenilhydrazine at room temperature for 15 min. DNP-derivatized proteins were separated by 12% polyacrylamide gel electrophoresis. DNP protein modifications were detected by rabbit anti-DNP antibodies according to the manufacturer’s protocol. Chemiluminescent detection was done by the Immobilon Western Chemiluminescent Horseradish Peroxidase (HRP) Substrate (Millipore) according to the manufacturer’s recommendations. The membrane was covered with a mixture of HRP Substrate Peroxide Solution and HRP Luminol Reagent (1:1) for 5 min at room temperature. Signals were visualized using the ChemiDoc Imaging System (Bio Rad, Hercules, CA, USA). Protein quantification was performed by densitometry using ImageJ software.

### 4.13. Statistical Analysis

Data are presented as the mean ± SD of values obtained in repeated experiments. For statistical analysis, one-way or two-way ANOVA followed by Tukey’s multiple comparison test was performed using the GrafPad Prism 8.0. Differences that reached a *p* value of less than 0.05 were considered statistically significant.

## Data Availability

The data and material that support the findings of this study are available from the corresponding author upon reasonable request.

## Supplementary Materials

The following supporting information can be downloaded at: https://www.mdpi.com/article/10.3390/ijms24032442/s1, Figure S1: Protein-Energy Interactions; Figure S2: Detection of *DSS1(I)* and *DSS1(V)* transcripts; Figure S3: Detection of the U6-26p promoter sequence from the CRISPR/Cas9 cassette in the T1 and T3 generation of the *dss1(I)*, *dss1(V)* mutants, and WT plants; Figure S4: Images of 12-day-old plants grown in MS vertical plates; Table S1: List of potential off-target site positions; Table S2: List of primers used to amplify the potential off-target sequences.
