# Supplementary material for: CRISPR/Cas9-Targeted Disruption of Two Highly Homologous Arabidopsis thaliana DSS1 Genes with Roles in Development and the Oxidative Stress Response
Source: Int J Mol Sci. 2023 Jan 26;24(3):2442. doi: 10.3390/ijms24032442 (PMC9916663; doi:10.3390/ijms24032442)
Supplement: Supplementary file 1 [file ijms-24-02442-s001.zip › ijms-2149846-supplementary.pdf]

Supplementary material

CRISPR/Cas9-targeted disruption of two highly homologous *Arabidopsis thaliana* DSS1 genes with roles in development and oxidative stress response

Ivana Nikolić[1], Jelena Samardžić[1], Strahinja Stevanović[1], Jovanka Miljuš-Đukić†[1], Mira Milisavljević[1], Gordana Timotijević[1]*

[1]Institute of Molecular Genetics and Genetic Engineering, University of Belgrade, Laboratory for Plant Molecular Biology, Vojvode Stepe 444a, 11042 Belgrade 152, Serbia

Corresponding author e-mail: timotijevic@imgge.bg.ac.rs

inter_2	inter_1	GBE	ACE	HB	ALIPH
**AT1G75990**	**dss1**	12.646667	4.775556	-1.813333	-0.833333
	**dss5**	37.482857	4.454286	-2.390000	-2.214286
**AT4G24820**	**dss1**	22.474000	4.867000	-1.342000	-0.500000
	**dss5**	43.506250	4.695000	-1.367500	-0.375000
**AT5G20000**	**dss1**	4.911818	7.035455	-1.869091	-0.727273
**ATS9**	**dss1**	3.304444	2.781111	-0.922222	-0.222222
	**dss5**	-1.987778	2.115556	-0.858889	-0.444444
**EERH5**	**dss5**	27.595833	5.945833	-1.957500	-1.416667
**EMB2719**	**dss1**	19.040000	6.229167	-2.310833	-0.791667
	**dss5**	-3.043333	3.983333	-0.510000	0.000000
**brca2B**	**dss1**	-11.611333	1.464000	-1.312000	-2.133333
	**dss5**	-11.460667	1.633333	-1.339333	-1.733333
**brca2IV**	**dss1**	7.463750	3.525000	-1.653750	0.000000
	**dss5**	5.709091	3.500909	-1.635455	-0.500000
**rpn10**	**dss1**	14.307059	4.128824	-1.640000	-0.558824
	**dss5**	1.425000	5.994375	-2.035625	-0.750000
**rpn12a**	**dss1**	19.924000	4.293000	-1.301000	-0.800000
	**dss5**	5.108182	3.272727	-0.903636	-0.500000
**rpn13**	**dss1**	20.591818	5.419091	-1.891818	-1.181818
	**dss5**	2.602727	1.583636	-0.997273	-0.545455

Figure S1: Protein-Energy Interactions. Calculations of the global binding energy (GBE), the unbound state to the complex (ACE), hydrogen and disulfide bonds (HB), and aliphatic interactions (ALIPH) between AtDSS1s and their protein partners.

Table S1: List of potential off-target site positions.

Gene ID	Target sequence	PAM	MM	Gene name	Position
**AT1G64750**	GACTGCTG [AAGTAGTAAAGA]	TGG		Deletion of SUV3 suppressor 1(I), DSS1(I)	Exon
**AT4G03400**	GACTG**T**TG [A**T**GTAG**A**AAAGA	TGG	3	GH3-like protein	Exon
**AT2G13100**	GACTGC**GC** [AAG**G**AG**C**AAAGA]	AGG	4	Glycerol-3-phosphate permease	Intergenetic region
**AT4G04960**	G**G**CT**C**CTG [AAGT**G**GT**T**AAGA]	CGG	4	Concanavalin A-like lectin protein kinase	Exon
**AT5G45010**	AGCTGTCG [AAGTGGTGAAGG]	TGG		Deletion of SUV3 suppressor 1(V), DSS1(V)	Intergenetic region
**AT4G06490**	**T**G**A**TGT**A**G [AAGTGGTGAAGG]	CGG	3	hypothetical protein (DUF3287)	Exon
**AT5G01170**	AGC**G**GT**G**G [**T**AGTGGTG**G**AGG]	CGG	4	OCTOPUS LIKE 1 hypothetical protein	Exon
**AT2G25800**	AG**T**TGTC**C** [**G**AGTGGTGA**T**GG]	AGG	4	Elongation factor Ts (DUF810)	Exon
**AT1G17500**	AGCTGT**GC** [AA**A**TGGTGAAG**C**]	TGG	4	ATPase E1-E2 type family protein	Exon
**AT4G14510**	AG**TA**GTCG [AAGTGGTGA**TGT**]	TGG	4	CRM domain protein CFM3b	Exon

Table S2: List of primers used to amplify the potential off-target sequences.

Gene ID	Primer name	Primer sequence	Product size (bp)
**AT4G03400**	I34f	5′- TGAGGAAATGGCTTGGGACT-3′	178
	I34r	5′- AGGGACAGAACAGTGATGGG -3′	
**AT4G04960**	I496f	5′- TTGGGTTAGCTCGGGTTCAT -3′	212
	I496r	5′- TCCCCAAACCCAATCCATCA -3′	
**T5G01170**	V117f	5′- CGGCGAGGAATAAGAGTTCG -3′	187
	V117r	5′- GCTATAGAGTGGCCGTGACT -3′	
**AT4G14510**	V145f	5′- GGGGTTATAAGTGGTGAGAACAG -3′	300
	V145r	5′- ACCGAACTCTTCTCAATTCTTCT -3′	
**AT2G25800**	V258f	5′- GTCACATTCGGCGACTTCAC -3′	168
	V258r	5′- ACAGCCGATGAGAGAGGTTT -3′	
**AT1G17500**	V175f	5′- TGCATAACTGTGGTGAACTCA -3′	169
	V175r	5′- CCTCCAATGCATACGTTAGGG -3′	

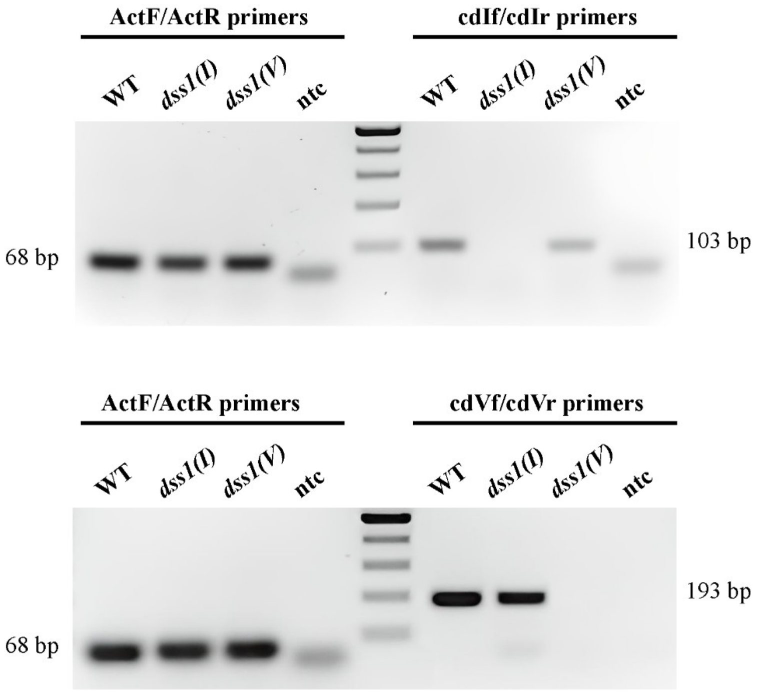

Figure S2: Detection of *DSS1(I)* and *DSS1(V)* transcripts. In Arabidopsis *dss1* mutant lines and WT plants, presence or absence of *DSS1(I)* transcripts was determined by PCR analysis using cdIf/cdIr primers (upper electrophoretogram) and presence or absence of *DSS1(V)* transcripts was determined by PCR analysis using cdVf/cdVr primers (bottom electrophoretogram). ActF/ActR primers used to amplify actin as a reference gene.

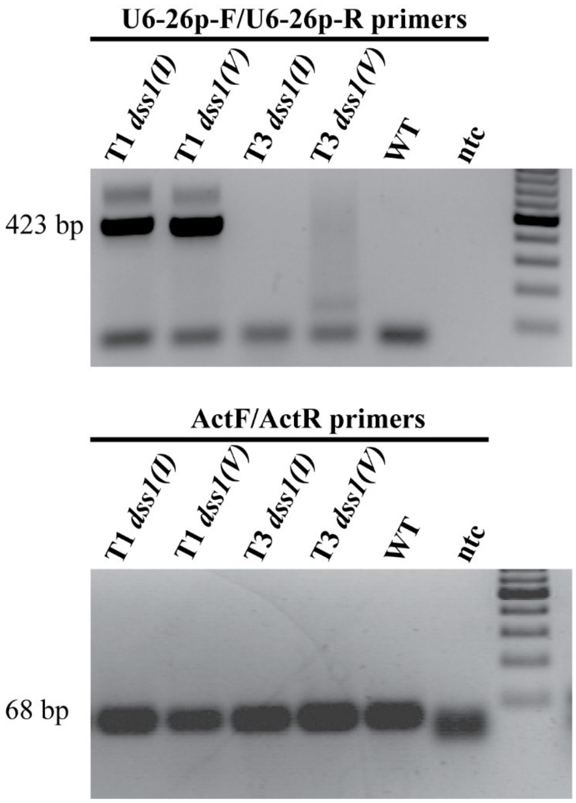

Figure S3: Detection of the U6-26p promoter sequence from the CRISPR/Cas9 cassette in the T1 and T3 generation of the *dss1(I)*, *dss1(V)* mutants, and WT plants. In Arabidopsis *dss1* mutant lines and WT plants, presence or absence of U6-26p promoter was determined by PCR analysis using U6-26p-F/ U6-26p-R primers (upper electrophoretogram. ActF/ActR primers used to amplify actin as a reference gene (bottom electrophoretogram.

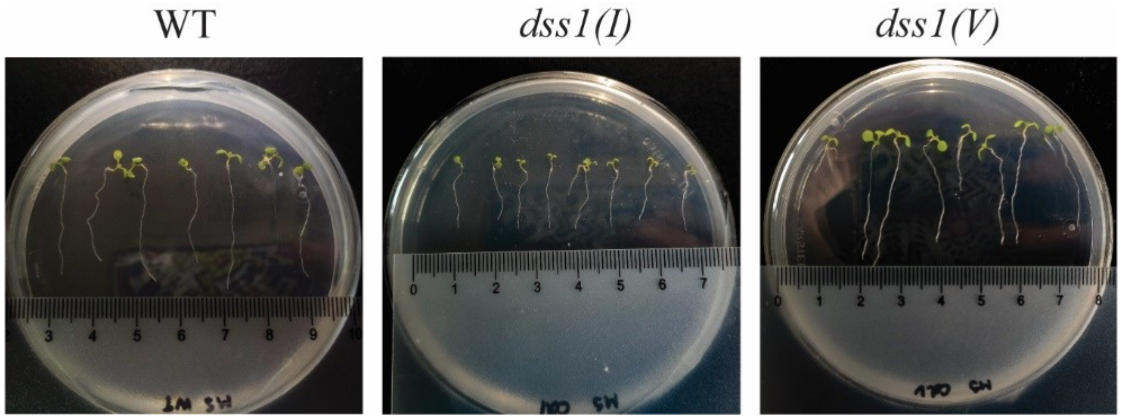

Figure S4: Images of 12-day-old plants grown in MS vertical plates. Photographs show representative WT, *dss1(I)*, and *dss1(V)* seedlings. A ruler on the photograph was used as a known distance in cm units for ImageJ setting measurement scale. Seedling length was measured by the tracing of each main root with the ImageJ freehand lines tool.
